# Cutting the edge between cancerogenesis and organogenesis of the pancreatic endocrine lineage allocation—comprehensive review of the genes *Synaptotagmin 13* and *533041C22 Rik* in epithelial-to-mesenchymal transition

**DOI:** 10.1007/s10555-020-09897-4

**Published:** 2020-05-24

**Authors:** Stefanie Julia Willmann

**Affiliations:** 1 TUM München, Arcisstraße 21, 80333 Munich, Germany; 2 Munich, Germany

**Keywords:** Pancreatic lineage allocation, Epithelial-to-mesenchymal transition, *Synaptotagmin 13*, *533041C22 Rik*, Cancerogenesis

## Abstract

In the past years, a multitude of studies has been published in the field of pancreatic organogenesis to interrogate the critical regulators of endocrine lineage segregation. Preliminary, transcription factors are guiding the transcriptional hierarchy of the endocrine specified cells, underpinning the importance of open chromatin formation. Signaling pathways either inhibit or accelerate the transcriptional landscape of pancreatic organogenesis. Thus, the fine-tuned process in the former pancreatic multipotent progenitors in the mechanism of lineage segregation needs to be elucidated more precisely for unraveling the temporal-spatial lineage-determining factors.

Previously, Willmann et al. described candidate gene regulators of lineage segregation during the secondary transition of pancreatic organogenesis. At embryonic stage (E) 12.5, the former multipotent pancreatic progenitor compartmentalizes into the acinar, ductal, and endocrine lineage. In the adult pancreatic gland, acinar cells secrete enzymes that are transported by the duct to the duodenum. In contrast, the endocrine cells are clustered within the acinar tissue in the Islets of Langerhans. These Islets of Langerhans consist of a subset of α, δ, ε, and PP cells and β cells, and the function of the α and β cells is predominantly described by regulating glucose homeostasis, contrary, the function of the additional subtypes in the Islets of Langerhans remains still unclear and is rather pointing to a supportive role for the α and β cells.

The essential wave of endocrine precursor cells emerges at E 14.5 out of the ductal cord-like structure in a process called epithelial-to-mesenchymal transition (EMT). This EMT is a reversible and incomplete process that includes significant intermedia states. As EMT is in focus in the field of cancer research, missense in endocrine lineage segregation is linking to a progression of pancreatic cancer, to be more precise in adenocarcinoma, e.g., meaning pancreatic ductal adenocarcinoma.

Thus, the previous review will further accelerate the understanding of EMT about endocrine lineage segregation, respective pancreatic ductal adenocarcinoma, and introduces factors previously only known for either lineage segregation or related in cancer disease into a complete picture.

## Background

The article summarizes a review of the current literature of pancreatic lineage allocation into the endocrine lineage, represented by the gene *Synaptotagmin 13*. Furthermore, a descriptive link of endocrine lineage allocation and cancerogenesis is described with a focus on *Synaptotagmin 13* and one more potential candidate *533041C22Rik*, which is incorporated in the discussion.

## Pancreatic lineage segregation—classical key regulators

The key transcription factor in pancreatic organogenesis can be considered the *Pdx1* gene, and lineage-tracing experiments demonstrate clearly that pancreatic progenitors are expressing the *Pdx1* result in the acinar, ductal, and endocrine cells in the mature pancreatic gland [3]. To which extend the upstream regulator of *Pdx1*, *Foxa2*, is involved in pancreatic organogenesis remains still elusive. Still, preliminary results may hint to a cooperatively driven transcriptional hierarchy of *Foxa2* and *Pdx1* in the fine-tuned pancreatic initiation at approximately embryonic day (E) 8.5 (unless stated otherwise, the embryonic stages refer to mouse embryonic development). Here precise regulation on a protein level further determines the lineages of the pancreatic progenitors and thereby pointing to a non-reversible established lineage of the different pancreatic progenitor cells instead of the speculated multipotency character in the pancreatic epithelium [21]. Thus, a subpopulation of a Pdx1^high^ directing the progenitors into ductal inherited cells consisting of ductal and endocrine progenitors, whereas the subpopulation of Pdx1^low^ inherits the acinar progenitor pool. This acinar progenitor pool is mainly characterized by the genes *Ptf1α*, *Cpa1*, and *Nr5a2* in which after the so-called first transition, at approximately E 9.5, these genes are expressed in the pancreatic epithelium and become later restricted in the secondary transition to the periphery, respective tip region of the evolving pancreas [12].

The main focus in pancreatic development is on endocrine lineage segregation as these cells will finally lead to the ß cell in the mature Islets of Langerhans. Thus, the ductal cord at E 14.5 persists of the bipotent progenitor’s preliminary marked by the gene *Sox9* located in the central region of the pancreas, the so-called trunk pattern. Also, recently published, *Sox9* is a critical player in ductal-derived endocrine cell differentiation in the pancreatic organogenesis, and the gene itself suggests not to contribute to endocrine cells in the mature gland. This trunk pattern inherits next to *Sox9* an *Ngn3*-transient cell population, thereby representing the endocrine progenitors. Lineage tracing experiments implicated for *Ngn3* as an essential helix-loop-helix transcription factor the necessity for the segregation of endocrine progenitors into the endocrine precursors as a downstream regulator of *Sox9* [2, 15, 16].

Furthermore, Lynn and Seymour proposed a cell-autonomous role for *Sox9* in *Ngn3* induction, which suggests a negative feedback-loop for co-related expression of *Sox9* and *Ngn3* [11, 17, 18]. This result may further determine the importance of the gene *Ngn3* in endocrine precursor segregation, especially regarding the upstream regulators *Sox9* and Pdx1^high^. The signal cascade for the regulation of *Ngn3* is still controversial, as there might be extrinsic and intrinsic signals that affect the proliferation of the endocrine lineage. Interestingly, signals from mesenchyme might not play a role in endocrine formation—as the proximity of the pancreatic epithelium to the mesenchyme accelerates the exocrine fate, whereas missing contact of the epithelium to the mesenchyme results in the endocrine lineage [10].

The entry of the endocrine precursors is marked by the family of *NKx6* transcription factor genes (Nkx6.1 and Nkx6.2). Thereby, Pdx1^high^ expression correlates with *Nkx6* transcription factor expression, suggesting an activation of *Nkx6.1* and *Nkx6.2* by a complex mainly characterized by an abundance of a certain level of the protein Pdx1. Also, phenotype analysis of mouse mutants does reveal an absent of ß cells for Nkx6.1 and respective α cells for Nkx6.2, these genes are not essential for proper pancreatic organ formation [4, 5, 14, 20]. Thus, these results may point that the endocrine precursors are already primed for the final lineage in the subpopulation of the immature Islets of Langerhans.

## Pancreatic ductal adenocarcinoma cancer factors—classical key regulators

The pancreatic ductal adenocarcinoma is a highly fatal disease because therapies against this tumor are ineffectively due to the metastatic stage, e.g., by a diagnosis of pancreatic ductal adenocarcinoma cancer factors (PDAC), the progression of the cancer is already spawned. Thus, the focus is on early diagnosis by the use of biomarkers for determining the state of occurrence of the disease. This neoplasia is described in the context of a progression model, wherein the ductal epithelium undergoes remodeling into PDAC through a series of so-called pancreatic intraepithelial neoplasia (PanIN), which are histologically defined precursors. Also, the PanINs are characterized by upregulated gene expression of *Her-2* and *K-ras* (PanIN-1A and PanIN-1B), *p16* (PanIN-2) and *p53*, and *DPC4* and *BRCA2* in PanIN-3; the review of Hruban and colleagues nicely depicts the essential pathway members and illustrates the model [6]. Contrary, the result only points to the descriptive role of overexpression of these genes in the context of neoplasia in the pancreatic ductal epithelium. Further data are lacking, especially given the mechanistically understanding.

## Pancreatic cancerogenesis and endocrine lineage segregation by mechanistic regulation through EMT

The classical model of EMT, already described in the context of cancerogenesis, suggests to depict the gatekeepers and hallmarks of pancreatic cancer progression. Moreover, as the mechanism of EMT is proposed in endocrine lineage segregation, deciphering novel factors may shed light into the intermediate states and the factors either stabilizing these states or accelerating the state of progenitors, respective precursors.

**Fig. 1:**
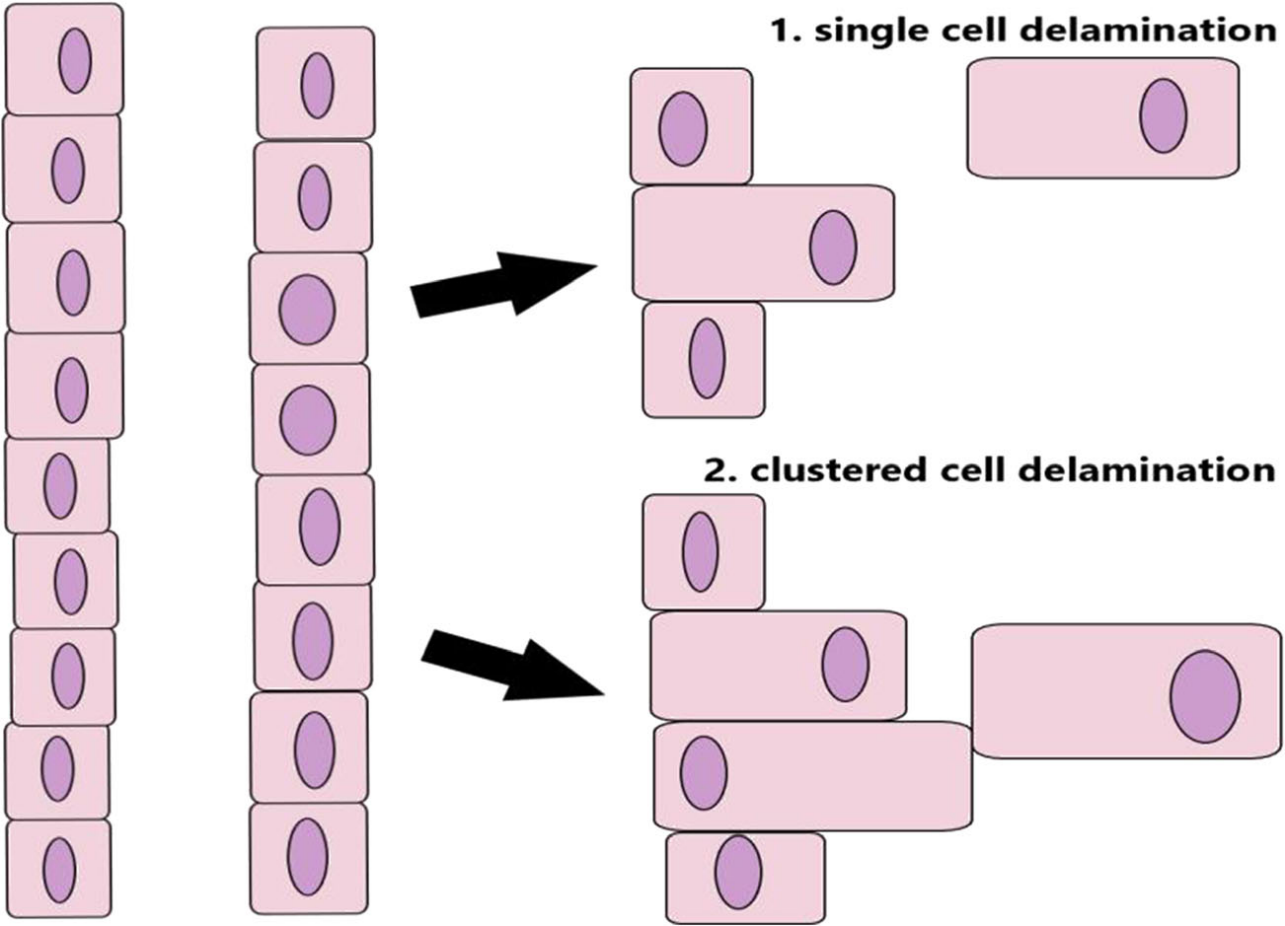
Model of EMT-induced cell delamination illustrated for the epithelial/endocrine cells as these cells leave the ductal cord to cluster into the acinar tissue of the pancreas by (1) single-cell delamination or by (2) clustered cell delamination

As the endocrine precursors emerge out of the ductal cord, the association of PDAC and endocrine lineage segregation about the mechanism EMT is clear by sight. We illustrate here current models in the process of delamination of cells out of the ductal cord. In this process, epithelial ductal inherited cells, either marked by a Ngn3^low^ or a Ngn3^high^ subpopulation stepwise, change the cell polarity into the acquisition of motility properties. The exact intermediate states of the formerly endocrine progenitors into endocrine precursors are poorly understood but may involve *Nkx6* transcription family members. Thus, we assume an *Nkx6.1*, respective *Nkx6.2* expressing precursor cells already represents an intermediate lineage determined endocrine cell. Notably, ductal neoplasia is characterized by the clonal growth of a single cell and later resulting in waves of clonal expansion represented here in Fig. 1 under (1) single-cell delamination. Under the perspective of the genetic progression model, at PanIN-3 stage, cells delaminate and will spawn later into the surrounding tissue, vessels, and neighboring tissue. Under the perspective of the occurrence of Nkx6 intermediate lineage-determining stages, an underlying EMT mechanism, as proposed by Fig. 1 under (2) clustered cell delamination, suggests to more likely represent the endocrine lineage segregation rather than single-cell delamination under Fig. 1 (1). Therefore, we propose a model in which the endocrine precursors are already pre-committed and subsequently leave the ductal cord in a temporal-spatial manner and congregate to the architectural structure of the pre-defined immature Islets of Langerhans in mice.

The first model instead suggests single-cell delamination in the context of cancer progression, respective PDAC in pancreatic organogenesis ((1) Single-cell delamination). Contrary, the second model, already proposed by Pictet et al. in 1972, illustrates stepwise delamination of the pancreatic progenitors and/or precursors and aggregation in of these cells in the pancreatic epithelium at lineage segregation during organogenesis between the E 14.5 and E 16.5.

## Regulators of pancreatic lineage segregation in cancerogenesis progression

**Fig. 2:**
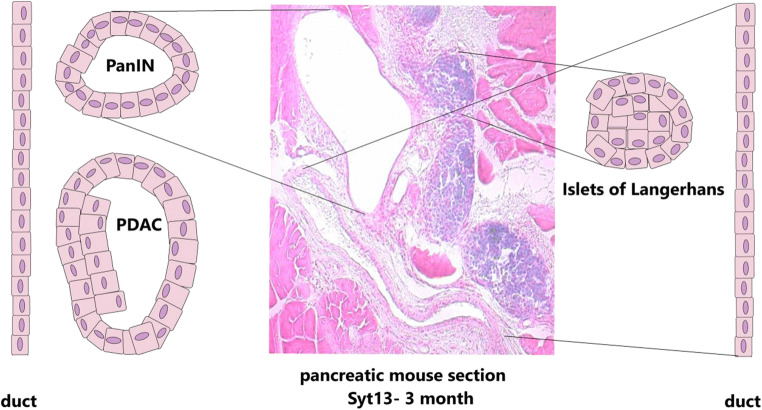
The *Syt13* gene trap mouse model represents a suitable model for the mechanism of EMT in the process of endocrine lineage segregation and the process of ductal lesions resulting in PanINs, respective PDAC

Recently, Roy and colleagues illustrated that *Pdx1* suggests regulating PDAC initiation and maintenance dynamically [13]. Preliminary, they investigated the tumor suppression and the oncogenic activity of *Pdx1* in the pancreatic exocrine gland. Furthermore, a subset of cancer cells undergoing EMT reveals reduced to none expression of the pancreatic epithelial marker *Pdx1*. Nevertheless, *Pdx1* may suggest being essential for the cancer progression in the pancreatic ductal lesion, either in acinar-to-ductal metaplasia (ADM) or PDAC. Thus, pointing to an intermediate state in the process of EMT and mechanistically different regulations of endocrine lineage segregation versus cancer progression. Taken the model from above (Fig. 1, (1) Single-cell delamination), single-cell delamination also illustrates the change in front-rear polarity by the depicted nucleus and thereby loss of the established epithelial polarity. Contrary, the model of endocrine precursors suggests at least a shift of polarity. However, the clustered cell delamination may hint to a still maintained elementary polarity for clustered cells to emerge out of the ductal cord into the immature Islets of Langerhans in an already defined architectural structure (Fig. 1, (2) Clustered cell delamination). In line with the previous illustrated results of *Pdx1* in PDAC are results of an upstream regulator of *Pdx1*. The gene *Foxa2* is a critical regulator of EMT, and thus loss-and-gain studies revealed suppressed expression in the process of EMT by break-down of adherents junctions via regulation of E-cadherin, but Foxa2 expression is re-activated in well-differentiated cancer cells.

Interestingly, the regulatory gene of acinar lineage segregation, *Ptf1α*, may play a role in oncogenic suppression of the PDAC, to be more precise in the PanIN of the ductal inherited cells and thus an expression of the gene *Ptf1α* is downregulated in invasive acinar PDACs [7]. Also, these results might suggest a partial EMT mechanism of the pancreatic epithelial cells in the process of lineage segregation and taken this hypothesis, the multipotency of the pancreatic epithelium is mainly guided through signaling pathways, e.g., Tgfβ or Wnt/PCP members as *Frizzled* (*Frz*) or *Dishevelled* (*Dsh*), activating or repressing junctional arrangement by use of the classically known lineage segregation factors. Therefore, the pancreatic epithelium in organogenesis does not per se have an established polarity, either in the acinar, ductal, or endocrine progenitor lineage, and with this in mind, the polarity determines the lineage rather than the transcriptional hierarchy.

Furthermore, the gene *Nr5a2*, after secondary transition specifically regional expressed in the tip region of the pancreatic epithelium, is described in the context of cancer progression as being upregulated and suggests to inhibiting EMT and thus, inhibit cancer progression in the pancreas [7] whereas Sox9 is directly linked to PDAC, but only present in a minor subpopulation of approximately 0.8% and not determined for the PDAC progenitor of PanINs [19]. Earlier published by Kopp et al. PanIN2/3 are represented by heterogenous *Sox9* expression and thereby associated with acinar-derived PDAC initiation. In *Kras*^*G12D*^ mice, *Sox9* was detected in the acinar tissue pointing to a concomitant of PDAC initiation by *Sox9* and *Kras*^*G12D*^ missense expression through suppression of the acinar lineage determinants. Also, several ductal genes are further promoted, but the suppressed acinar tissue is not capable of being reprogrammed into ductal progenitors. As a result, an ADM does not necessarily depend on *Sox9* expression, but *Sox9* suggests to be essential in the stages of the progenitor stages of the ADM, the PanINs [8]. With this in mind, *Ngn3*, downstream of *Sox9*, is described only in the context of being repressed by *Zinc Finger E-Box Binding Homeobox 1* (*Zeb1)*, whereas *Zeb1* initiates a tumorigenic phenotype, likely because a partial EMT is induced. Further experimental designs in regard to *Ngn3* as a potential player in the initiation of PanINs will shed light on the partial EMT mechanism and the differences between endocrine versus cancerogenesis in this mechanism [22].

The family of *Nkx6* genes is rarely described in the context of cancerogenesis. Cervical cancer cell lines are used for determining the absence of *Nkx6.1* accompanied by the absence of the junctional polarity established by E-cadherin. Furthermore, mesenchymal properties, as stated by *Vimentin* expression, are downregulated in *Nkx6.1* stable transfected Hela and/or SiHa cells with the conclusion by the authors of repressing cancer invasion by accelerating expression of epithelial markers [9]. We further suggest that *Nkx6.1* instead not suppresses EMT but authorizes a partial EMT, which differs from the classical-known mechanism of EMT regarding cancerogenesis. Thus, we refer to the different models proposed for the mechanism of EMT and suggest only a partial loss of polarity in the process of endocrine formation. Further experiments would be sufficient for prefiguring the involvement of the mechanism of EMT in endocrine progenitors as *Ngn3* and/or subsequent endocrine precursors as *Nkx6.1*, respective *Nkx6.2*.

## Conclusion

### Further perspectives of novel putative lineage segregation factor and the relation to PDAC/PanIN

Recently published, *Synaptotagmin 13* suggests being involved in the process of endocrine lineage segregation, as preliminary results point to concomitant expression of *Foxa2*, in particular, Foxa2^high^, respective Pdx1^high^, and *Syt13* [21]. The precise function of *Syt13* remains unclear, and *Syt13* may instead be involved in vesicle-mediated transport, e.g., as a scaffold protein in trafficking within a cell, because typical exocytotic properties of the *Synaptotagmin* gene in the amino acid structure are lacking in *Syt13*. As observed in the pancreatic paraffin section of 3-month mutant mice, *Syt13* hint to the process of cell delamination in the mechanism of EMT. By using a *gene trap* allele, including an incorporated *lacZ*-reporter cassette in the floxed allele of the mutant mice, *Syt13* expression can be followed by staining for *β-galactosidase* (kindly provided by EUCOMM). Thus, the reporter construct illustrates in Fig. 2, the representative staining in blue color in the Islets of Langerhans, identified by the confined cells by dense aggregation. Furthermore, the pancreas illustrates pancreatic lesions, categorized into PanIN 2, as the cells lining the lesion represent a monolayer. Taken these preliminary results, the *Syt13* mutant mouse model suggests being a suitable model for mimicking the mechanism of EMT in endocrine lineage segregation and the process of cancerogenesis, in particular, the PanINs and in later stages, the PDAC-derived tumors. The partial EMT in both processes may be investigated more precisely for evaluating the different signaling factors for either choice and, in particular, for the partial EMT.

On the left side, PanIN and PDAC illustrated as ductile-derived cancerogenesis lesions of the pancreas. A monolayer characterizes the PanIN, wherein the cells of the monolayer lining the PanIN have a nucleus in the still orientated stable polarity complex. Contrary, the PDAC illustrates a multilayer of cells, wherein the cells are representing nuclei differentially distributed within the cells, and thus, invasive characteristics. In the pancreatic section of 3-month mice of the *Syt13* mutant, the PanIN is indicated by the black lines.

On the right side, the ductal-derived endocrine precursors are orientated in a pre-defined manner in the Islets of Langerhans and on the pancreatic section of the 3-month mice of the *Syt13* mutant indicated by the black lines. Furthermore, the duct is indicated by the black lines on the paraffin section. The pancreatic section illustrates that the pancreatic lesion and the Islets of Langerhans are ductal-derived by a mechanism proposed as EMT. Furthermore, a factor involved in cancerogenesis and endocrine progenitor, respective precursor lineage segregation, suggests being *Syt13*.

In addition, the gene *533041C22* Rik may be a promising target for further elucidating factors essential for partial EMT. The gene itself is already described in the context of the gene *maba-1* (also known as *KIAA1324*), and the expression level of *maba-1* suggests of guiding metastasis and tumor progression in breast and lung cancer cell lines [1]. Also, knockdown of *maba-1* on an mRNA level and an antibody inhibits progression of cancer, e.g., the proliferation of HCT116 colon cancer cells (EPO Global Dossier US 64783309), and thus, pointing to a role of maba-1 in remodeling an epithelial cell into a cell capable of being invasive and migratory. Also, preliminary results may hint to *maba-1* as a factor in endocrine lineage segregation, which as described above, could be a suitable factor for determining the partial EMT, especially in regard of the convention and the differences of the mechanism of EMT in view of endocrine lineage segregation and cancer progression.

Thus, two putative novel genes in endocrine lineage segregation reveal a direct and inevitable link to cancerogenesis. Furthermore, as endocrine progenitors are inherited in the ductal cord during pancreatic organogenesis, these two putative endocrine lineage segregation factors may play a role in the mechanism of EMT and thereby suggest to be investigated closer in regard to the key partial EMT which differentiates the mesenchymal-derived cancerogenesis cell from the endocrine precursor cell.

## Data Availability

The datasets used and/or analyzed during the current review are available from the corresponding author and are made released to the public under URN: urn:nbn:de:bvb:91-diss-20160711-1278135-1-9.

## Abbreviations

ADM: acinar-to-ductal metaplasia
BRCA2: BRCA2 DNA Repair Associated
Cpa1: Carboxypeptidase A1
DCP4: Decapping Enzyme 4
Dsh: Dishevelled
E: embryonic stage
EMT: epithelial-to-mesenchymal transition
Foxa2: Forkhead box 2
Frz: Frizzled
Her-2: *Erb-B2 Receptor Tyrosine Kinase 2*
K-ras: *KRAS Proto-Oncogene*
Maba-1: *KIAA1324*
Ngn3: Neurogenin 3
Nkx6: NK6 Homeobox 2
Nr5a2: Nuclear Receptor Subfamily 5 Group A Member 2
P16: protein 16
P53: protein 53
PanIN: pancreatic intraepithelial neoplasia
PCP: planar cell polarity
PDAC: pancreatic ductal adenocarcinoma
Pdx1: pancreatic and duodenal homeobox 1
PP: pancreatic polypeptide cells
Ptf1α: Pancreas Associated Transcription Factor 1a
Sox9: SRY-Box Transcription Factor 9
Syt13: Synaptotagmin 13
Tgfβ: transforming growth factor beta
Wnt: wingless
Zeb1: Zinc Finger E-Box Binding Homeobox 1
α: alpha cells
β: insulin-producing cells
δ: delta cells
ε: etha cells
